# Airway microbiome composition correlates with lung function and arterial stiffness in an age-dependent manner

**DOI:** 10.1371/journal.pone.0225636

**Published:** 2019-11-26

**Authors:** Shuen Yee Lee, Micheál Mac Aogáin, Kai Deng Fam, Kar Ling Chia, Nur A’tikah Binte Mohamed Ali, Margaret M. C. Yap, Eric P. H. Yap, Sanjay H. Chotirmall, Chin Leong Lim

**Affiliations:** Lee Kong Chian School of Medicine, Nanyang Technological University, Singapore; University of Alabama-Birmingham, UNITED STATES

## Abstract

**Objective:**

To investigate age-associated changes in airway microbiome composition and their relationships with lung function and arterial stiffness among genetically matched young and elderly pairs.

**Methods:**

Twenty-four genetically linked family pairs comprised of younger (≤40 years) and older (≥60 years) healthy participants were recruited (Total *n* = 48). Lung function and arterial stiffness (carotid-femoral pulse wave velocity (PWV) and augmentation index (AIx)) were assessed. Sputum samples were collected for targeted 16S rRNA gene amplicon sequencing and correlations between microbiome composition, lung function and arterial stiffness were investigated.

**Results:**

Elderly participants exhibited reductions in lung function (FEV1 (*p<*0.001), FVC (*p*<0.001) and percentage FEV1/FVC (*p* = 0.003)) and a 1.3–3.9-fold increase in arterial stiffness (*p<*0.001) relative to genetically related younger adults. Elderly adults had a higher relative abundance of Firmicutes (*p* = 0.035) and lower relative abundance of Proteobacteria (*p* = 0.014), including specific genera *Haemophilus* (*p* = 0.024) and *Lautropia* (*p* = 0.020) which were enriched in the younger adults. Alpha diversity was comparable between young and elderly pairs (*p*>0.05) but was inversely associated with lung function (FEV1%Predicted and FVC %Predicted) in the young (*p* = 0.006 and *p* = 0.003) though not the elderly (*p* = 0.481 and *p* = 0.696). Conversely, alpha diversity was negatively associated with PWV in the elderly (*p* = 0.01) but not the young (*p* = 0.569). Specifically, phylum Firmicutes including the genus *Gemella* were correlated with lung function (FVC %Predicted) in the young group (*p* = 0.047 and *p* = 0.040), while Fusobacteria and *Leptotrichia* were associated with arterial stiffness (PWV) in the elderly (both *p* = 0.004).

**Conclusion:**

Ageing is associated with increased Firmicutes and decreased Proteobacteria representation in the airway microbiome among a healthy Asian cohort. The diversity and composition of the airway microbiome is independently associated with lung function and arterial stiffness in the young and elderly groups respectively. This suggests differential microbial associations with these phenotypes at specific stages of life with potential prognostic implications.

## Introduction

Ageing is an identified risk factor for several chronic health conditions, including lung and cardiovascular disease (CVD), that exhibit disproportionate susceptibility, morbidity and mortality in older individuals [1, 2]. During ageing, subclinical lung function decline [3, 4], vascular endothelial dysfunction and arterial stiffening occur in the healthy population [5–7]. In older individuals above age 40 [8], parameters of lung function are inversely associated with arterial stiffness parameters, even after adjustment for cardiovascular risk factors, smoking history and lung disease [9, 10]. Reduced pulmonary function is independently associated with precursors for CVD including arterial stiffness, regardless of age, sex or anthropometry [8–12] and is an independent risk factor for cardiovascular morbidity and mortality [13]. Pulmonary function also predicts the development of atherosclerotic plaques and is a strong risk factor for arterial stiffness, suggesting a causal relationship between the decline in lung function and arterial stiffness [11, 12, 14].

While evidence supports the relationship between declining lung function, arterial stiffness and subsequent onset of CVD [13], the mechanisms underlying these associations are not known. Possible mechanisms include inflammation, as arterial stiffness is markedly increased in patients with chronic obstructive pulmonary disease (COPD), a condition characterized by airflow obstruction and inflammation [15–17]. While systemic inflammation, weight, smoking history, hypercholesterolemia, hypertension and diabetes have all been implicated, they do not fully account for the relationship between pulmonary function and arterial stiffness, suggesting that other factors could be involved [9, 13, 18].

The lung microbiome encompasses the collective genetic information of all microorganisms that colonize the lung and differs between healthy and diseased individuals [19]. Lower microbiome diversity and the presence of specific microbial taxa are associated with decreased lung function in pathogenic disease states [19, 20]. In healthy individuals, the lung microbiome comprises a community of low density and transiently present microorganisms, whereas pathogenic organisms predominate in disease [21–23]. While the effects of ageing on the lung microbiome remain to be established, the gut microbiome has been found to influence healthy ageing through alteration of inflammatory markers, diet and immune response [24–26]. Furthermore, age-associated alterations in immune function may affect the microbiome (or vice versa), generating low-grade inflammation, a key contributor to arterial stiffness and declining lung function [27]. An emerging question is whether the observed immunopathological associations between gut microbiota and disease also hold true at other anatomical sites such as the lung, where the microbiome may underpin age-associated changes in lung function with a consequent impact on arterial stiffness.

Here we investigated the effect of age on the airway microbiome in healthy individuals of Asian origin. Age-related changes in airway microbiota, between young and elderly family pairs, were assessed for their association with normative age-associated changes in lung function and arterial stiffness, as potential prognostic indicators of cardiorespiratory health.

## Methods

### Subjects

Twenty-four genetically linked family pairs (parent and child) were prospectively recruited. Younger participants were 22–39 years old and older participants were 60–71 years old. All participants were non-smokers, not on long-term inhaled medication, had no history of chronic respiratory diseases and resided in Singapore over the 10–12-month period preceding recruitment. None of the female participants were on oral contraceptives or hormone replacement therapy. The participants were recruited through posters, social media and community outreach events. All the participants were briefed on the nature and risks involved in the study and their rights to withdraw their participation without obligation before giving their written informed consent to participate in the study. The procedures in the study were approved by the institutional review board of Nanyang Technological University, Singapore (IRB-2017-12-010).

### Study design and procedures

Participants abstained from caffeine and dietary supplementation for 24 h and kept to their regular diet and sleep routines, as well as refrained from strenuous physical activity for 48 h prior to their visit to the laboratory. Participants arrived at the laboratory between 0900 h and 1000 h after having consumed a light meal >2 h before the trial. They had their blood pressure (BP) measured and declared that they were well for participation. Nude body weight was measured using an electronic scale (SECA, Hamburg, Germany) and height was measured using a stadiometer (SECA). Body mass index (BMI) was calculated as body weight (kg) divided by height (m) squared. Waist circumference (WC) was recorded using a tape measure (SECA) placed snugly at the waistline, midway between the lowest ribs and iliac crest in a standing position [28], recorded to the nearest 0.1 cm in triplicate and averaged from the measurements.

### Sputum collection and processing

Participants rinsed their mouth with water, before careful instructions were given on how to produce sputum from a deep cough, using the huff cough method [29]. Participants took three deep maximal breaths, before coughing as hard and as deeply as possible in a standing position. Sputum was collected by spontaneous expectorate from a deep cough (0.5–2 g) into sterile containers and were visually inspected before placing on ice immediately. Sputasol (ThermoFisher Scientific, Massachusetts, United States) was added into the sputum container in equal amounts. The sputum sample was then shaken at 200 rpm at 37 °C for 15 min. RNALater solution (ThermoFisher Scientific, Massachusetts, United States) was added and the sample stored in 1 mL aliquots at –80 °C. Saliva samples were assessed in 6 participants (3 matched pairs) to allow comparative assessments between oral and lung sampling. Prior to sputum collection, participants rinsed their mouth with water and saliva samples (5 mL) were collected into a 50 mL sterile tube by allowing saliva to accumulate before expectoration and the samples were placed on ice immediately [30]. Saliva samples were aliquoted (500 μL) and RNALater solution (1 mL) added and samples stored at –80 °C.

### Spirometry

Lung function measurement was conducted using a portable spirometer (Spirolab, MIR Medical International Research SRL, Italy), according to international guidelines [31]. Lung function parameters were determined as the forced expiratory volume in 1 s (FEV1), forced vital capacity (FVC) and percentage of FEV1/FVC. The FEV1 and FVC percentage predicted were calculated in the Spirolab software by the ATS/ERS standards, depending on ethnicity group [32].

### Arterial stiffness measurement

An indirect and non-invasive measure of arterial stiffness was determined by the SphygmoCor XCEL device (AtCor Medical Pvt, Ltd, Sydney, NSW, Australia). The arterial stiffness parameters included carotid-femoral arterial pulse wave velocity (PWV) and augmentation index (AIx). The participants rested quietly in a supine position at room temperature for 15 min before measurements of arterial pulse pressure waveforms, by an inflated cuff at the brachial artery. SphygmoCor system calculates the central aortic augmentation pressure (AP) by subtracting the pressure at the first systole resulting from the return of the reflected wave from the systolic pressure. The AIx was calculated as the ratio of AP to pulse pressure [33]. PWV was measured simultaneously with pressure transducers, by acquiring a carotid pulse by applanation tonometry and a femoral pulse by volumetric displacement, within a cuff around the upper thigh (femoral artery) [34, 35]. The pulse waves were captured electronically on a computer using the SphygmoCor system and accepted by the system after consistent high-quality waveforms were measured. The average of approximately 3–5 measurements were taken.

### DNA extraction and 16s rRNA gene sequencing

Sputum and saliva samples were thawed on ice and homogenised using glass beads (1mm, Sigma-Aldrich) using a bead mill homogeniser (VWR). DNA was purified using the Roche High-pure PCR Template Preparation Kit (Roche) as previously described [36]. Blank extractions from sterile PBS were also performed and served as negative extraction controls. All extracted samples were quantitated using the Qubit dsDNA High Sensitivity (HS) Assay Kit (Invitrogen) and visually assessed for integrity by electrophoresis on a 0.8% agarose gel.

### Sequence data processing and taxonomic assignment

Using extracted sputum DNA samples, libraries for targeted amplicon sequencing were prepared following the “16S Metagenomic Sequencing Library Preparation” guide (Part# 15044223 rev. B, Illumina) [37]. This 300-bp paired-end sequencing protocol was performed on a MiSeq sequencing platform (Illumina) at the Lee Kong Chian School of Medicine (LKCMedicine), Singapore. Targeted amplicon sequences were analysed using the 16S metagenomics tool (version 1.0.1; Illumina), using as a taxonomic database the Illumina-curated version of the May 2013 Greengenes Consortium Database (greengenes.secondgenome.com) release and the Ribosomal Database Project (RDP) Classifier as the classification algorithm. Control samples from negative PCR and blank DNA extractions were also sequencing and assessed to detect potential contaminants using the decontam statistical package [38].

### Statistical analysis

All statistical analyses were performed using Statistical Package for Social Sciences, version 23 (SPSS, Inc., Chicago, IL) and R version 3.3.3 (R Foundation for statistical computing, Vienna, Austria). Numerical variables are presented as mean (standard deviation, SD) in text and figures unless otherwise stated. The participant characteristics were analysed using paired t-test to assess potential differences between younger and older groups. Between group differences for relative abundance of both saliva and sputum microbiome phyla and genera, as well as alpha diversity (Shannon, Simpson and inverse Simpson index) were also assessed using paired t-test. Due to the skewed distribution of data, a square root transformation was performed for relative abundances of *Firmicutes*, *Proteobacteria*, *Haemophilus* and *Lautropia*, and a logarithmic transformation was performed for PWV to achieve a normal distribution for paired t-test. Pearson’s and Spearman’s correlation was used to evaluate associations between two variables that were either normally distributed or not normally distributed respectively, including any sub-group analyses. Principal coordinate analysis (PCoA) was used to assess the beta diversity and overall lung microbiome composition with age. PCoA plots for were generated using the first two principal coordinates according to age categories (young *vs*. elderly), as well as according to sample type (saliva *vs*. sputum). The ‘vegan’ R package (version 2.4–5) was used to calculate alpha diversity and implement ‘adonis’, which uses permutational multivariate analysis of variance, to test for statistical significance of association of overall beta diversity of lung microbiome composition with age. Bray Curtis distances between paired and unpaired young-elderly comparisons were assessed using the “dist_groups” function from the R package ‘udist’. All the results in the tables and figures are presented using the non-transformed data. A value of *p* < 0.05 was considered statistically significant.

## Results

### Age-associated changes in physiology of study cohort

#### Anthropometry and blood pressure

The demographics of healthy participants in our study are presented in Table 1. Despite no difference in weight (*p* = 0.734), older adults were shorter in stature (*p* < 0.001) and had higher BMI (*p* = 0.03) than the younger adults. Waist circumference was also greater in older than younger adults (*p* = 0.03). Compared with younger adults, older adults also had higher systolic BP (*p* = 0.002), although diastolic BP was similar between groups (*p* = 0.213) (Table 1).

**Table 1:** Mean and (standard deviation (SD)) of participant demographics.

	Young	Elderly	*p* value
*n*	24	24	
Sex *n* (M/F)	11/13	6/18	
Age (years)	29 (5)	63 (2)	**<0.001**
Weight (kg)	60 (12)	59 (13)	0.734
Height (cm)	166 (9)	157 (9)	**<0.001**
BMI (kg/m^2^)	21.7 (3.0)	23.9 (3.5)	**0.03**
WC (cm)	72.7 (9.4)	79.5 (11.7)	**0.03**
Systolic BP (mmHg)	112 (10)	123 (16)	**0.002**
Diastolic BP (mmHg)	71 (8)	74 (9)	0.213
FEV1 (L)	3.4 (0.7)	2.0 (0.6)	**<0.001**
FVC (L)	3.9 (1.0)	2.6 (0.6)	**<0.001**
FEV1/FVC (%)	86 (6)	79 (8)	**0.003**
FEV1 (% Predicted)	97 (14)	88 (15)	0.063
FVC (% Predicted)	96 (14)	91 (17)	0.361
FEV1/FVC (% Predicted)	100 (7)	97 (10)	0.157
Pulse Wave Velocity (PWV) (m/s)	6.5 (1.1)	8.4 (1.7)	**<0.001**
Augmentation Index (AIx)	8.7 (9.9)	24.6 (8.1)	**<0.001**

Paired t-test comparison between younger and older groups for anthropometry, blood pressure, lung function and arterial stiffness. Significant *p* values are indicated by bold typeface.

#### Lung function and arterial stiffness

Lung function parameters including FEV1 (*p* < 0.001), FVC (*p* < 0.001) and FEV1/FVC (*p* = 0.003) were all reduced in older adults compared with their younger family pairs (Table 1). Compared with younger adults, older adults had 41% or 1.4 L lower FEV1, 33% or 1.3 L lower FVC and 7% lower percentage of FEV1/FVC. However, adjusted FEV1 (%Predicted) and FVC (%Predicted) were comparable between younger and older adults (*p* = 0.063 and *p* = 0.361) suggesting lower lung function values were reflective of natural lung function decline in our healthy elderly cohort. The percentage of predicted value for FEV1/FVC percentage in younger and older family pairs was comparable (*p* = 0.157), and the values were all within healthy ranges [39]. Compared with younger adults, older adults had increased arterial stiffness, indicated by PWV and AIx (all *p* < 0.001). In both younger and older adults, arterial stiffness indicated by PWV and AIx were also within healthy ranges [35], and were 1.3-fold and 3.9-fold higher in older than in younger adults respectively (Table 1). Lung function was inversely associated with arterial stiffness. However, when age was adjusted for, in a partial correlation model, the associations between FEV1, FVC and percentage FEV1/FVC, with arterial stiffness parameters AIx and PWV were not significant (S1 Table).

### Age-associated changes in lung microbiome composition

#### Composition of lung microbiome from sputum samples in healthy individuals

**Fig 1:**
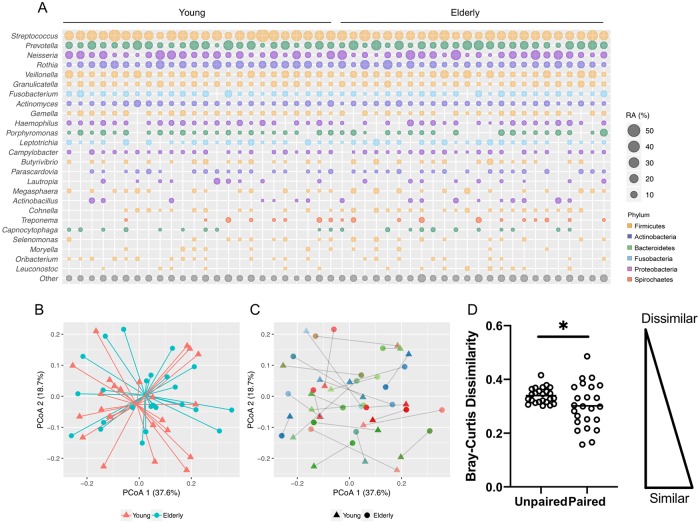
Lung microbiome composition in genetically paired young and elderly subjects. (A) Lung microbiome composition as detected by 16s rRNA gene profiling. Average relative abundance (RA) for the most representative taxa (present at >1%) is illustrated by circle size. Colour denotes phylum level membership. (B) Principle co-ordinate analysis (PCoA) of Bray-Curtis distance between microbiome profiles observed in young (red triangles) and elderly (turquoise circles) cohorts. (C) PCoA of microbiome profiles indicating linked young (triangles) and elderly (circles) participant pairs. Grey lines illustrate the distance between each paired sample which also share the same colour (D) Comparison of bray-curtis distance between PCoA points illustrating the reduction in dissimilarity observed between paired, genetically-related participants. * = *p* < 0.05.

The main phyla and genera identified in the airway microbiome of younger and older adults are shown in Fig 1. Sputum samples from healthy individuals, both young and elderly, consisted mainly of microbes from phylum Firmicutes (46%), Proteobacteria (16%), Bacteroidetes (16%), Actinobacteria (14%) and Fusobacteria (6%). The main genera detected were *Streptococcus* (24%), *Prevotella* (12%), *Neisseria* (10%), *Rothia* (8%), *Veillonella* (7%), *Granulicatella* (4%), *Fusobacterium* (4%), *Actinomyces* (3%), *Gemella* (2%), *Haemophilus* (2%), *Porphyromonas* (2%), *Leptotrichia* (2%), *Campylobacter* (1%) and *Lautropia* (1%) (Fig 1A). Comparison of saliva samples (total *n* = 6) suggested a high degree of overlap between the microbiota of healthy sputum and saliva with no differences in alpha- (Shannon diversity index (SDI) *p* = 0.304) or beta-diversity (*p* = 0.582) (S1 Fig). However, sputum samples, which are commonly used to represent the lung microbiome, exhibited subtle differences in specific taxa including higher relative abundance of *Granulicatella* (4.3% *vs*. 3.0%, *p* = 0.031), lower relative abundance of *Leptotrichia* (0.8% *vs*. 1.4%, *p* = 0.013) and lower relative abundance of *Corynebacterium* (0.7% *vs*. 0.1%, *p* = 0.031) (S1 Fig).

#### Comparison of lung microbiota present in genetically related young and elderly family pairs

**Fig 2:**
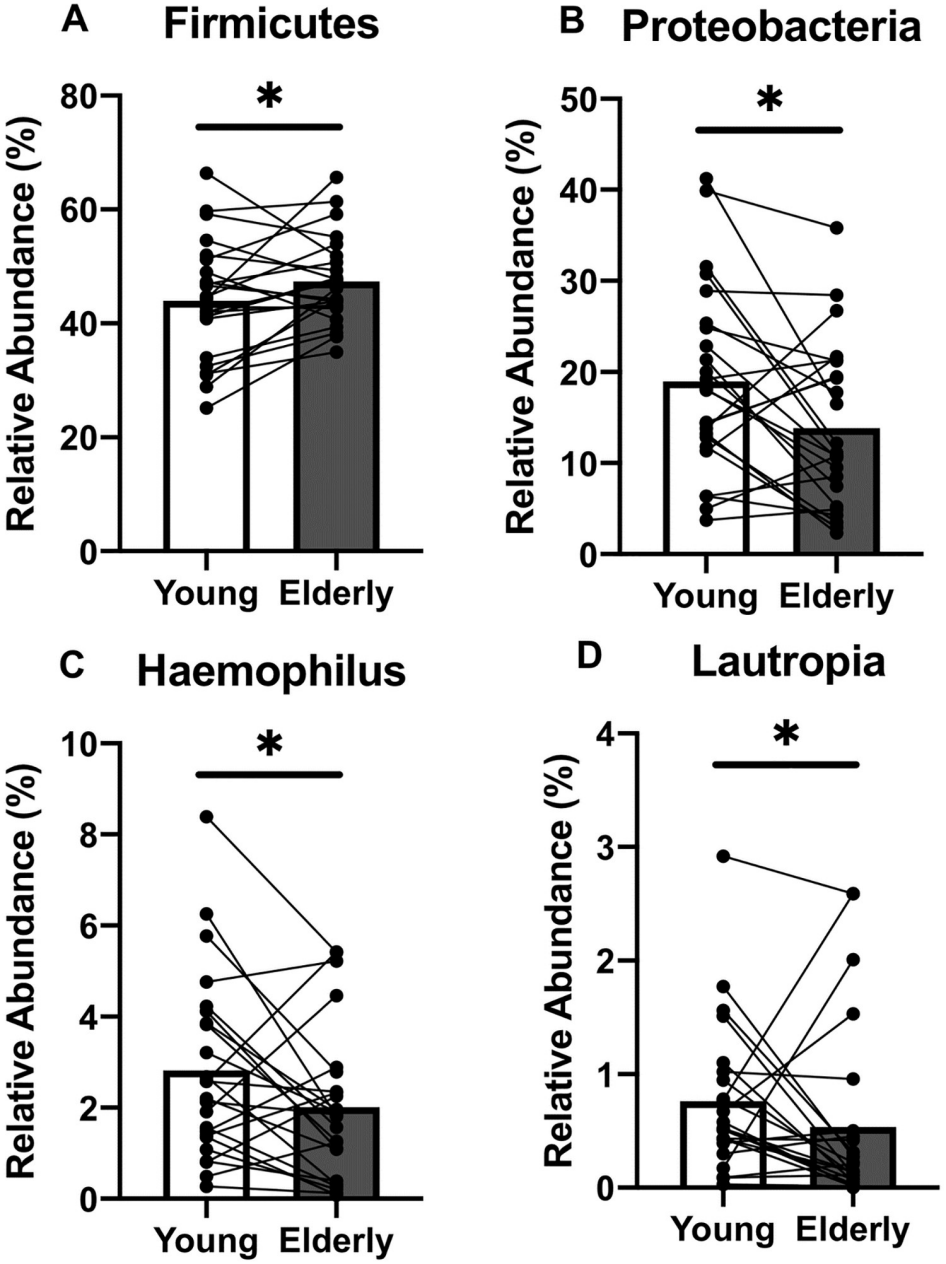
Differentially abundant taxa are observed in genetically paired young and elderly subjects. At the phylum level, differences in Firmicutes (A) and Proteobacteria (B) are observed between young (white bars) and elderly (grey bars) paired groups, with elderly adults exhibiting higher relative abundance of Firmicutes, and lower relative abundance of Proteobacteria than young adults. At the genus level, *Haemophilus* (C) and *Lautropia* (D) are differentially abundant, with lower relative abundance in elderly than in younger adults. Black connecting lines indicate young-elderly pairs. * = *p* < 0.05.

In order to allow some degree of control for genetic confounders, we assessed the airway microbiota in related young-elderly family pairs. PCoA analysis revealed that the sputum microbiome of younger and older adults was more similar when analysed as matched family pairs. Here, the measured Bray Curtis distance of matched young-elderly pairs was lower (equating to more similar microbiome profiles) when compared to the average distance between all other possible un-matched young-elderly comparisons (*p* = 0.036) (Fig 1C and 1D). Paired analysis showed that older adults generally had higher relative abundance of Firmicutes (47.4% *vs* 43.9%, *p* = 0.035) and lower Proteobacteria (13.9% *vs*. 19.0%, *p* = 0.014) with lower average relative abundance of Proteobacteria including *Haemophilus* (2.0% *vs*. 2.8%, *p* = 0.024) and *Lautropia* (0.5% *vs*. 1.1%, *p* = 0.020) (Fig 2). No significant differences in alpha diversity measures were observed between young and elderly groups (S2 Fig). Beta-diversity analysis also showed no difference between age groups in overall lung microbiome composition (*p* = 0.420, Fig 1B).

### Association of lung microbiome with lung function and arterial stiffness

#### Association of lung microbiome alpha diversity with lung function and arterial stiffness

**Fig 3:**
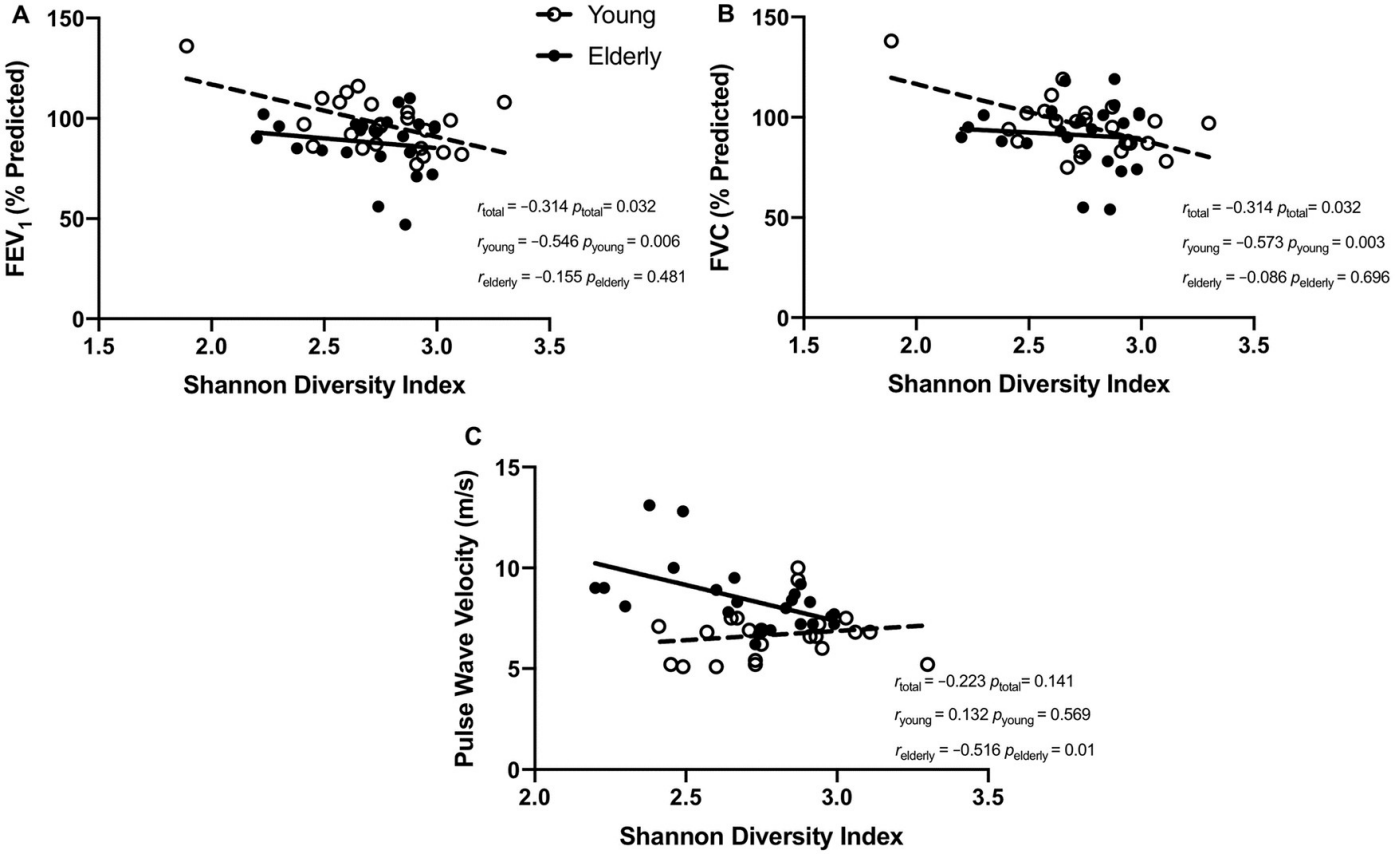
Physiological correlates of microbiome alpha-diversity in young and elderly subjects. Sub-group correlations between the Shannon diversity index of the lung microbiome and lung function parameters (A) FEV_1_ (% Predicted), (B) FVC (% Predicted) as well the arterial stiffness parameter (C) pulse wave velocity. Young and elderly groups are indicated by unfilled and filled circles respectively. The line of best fit is indicated with a broken line for the young population and with a continuous line for the elderly. Correlation coefficients and associated *p* values are indicated for the total population (*r*_total_), the young sub-group (*r*_young_) and the elderly sub-group (*r*_elderly_).

Alpha diversity of the lung microbiome was associated with lung function in an age-dependent manner. While SDI was associated with both FEV1 (%Predicted) and FVC (%Predicted) in the cohort as a whole (all *r* = –0.314, *p =* 0.032), the association was driven by the younger group (Fig 3A and 3B). In the younger group, FEV1 (%Predicted) and FVC (%Predicted) were both associated with SDI (*r* = –0.546, *p =* 0.006 and *r* = –0.573, *p =* 0.003) (Fig 3A and 3B). However, in the older group, the association between FEV1 (%Predicted) and FVC (%Predicted) with SDI were not significant (*r* = –0.155, *p =* 0.481 and *r* = –0.086, *p =* 0.696) (Fig 3A and 3B). In the young group, Shannon diversity was also associated with absolute FVC (*r* = –0.411, *p =* 0.031) (Table 2). Conversely, alpha diversity of the lung microbiome was correlated with arterial stiffness (PWV) in the older but not the younger group (*r* = –0.516, *p =* 0.01 *vs r* = 0.132, *p =* 0.569) (Fig 3C). Correlations with inverse Simpson index showed similar associations as SDI (Table 2).

**Table 2:** Correlation matrix comparing lung microbiome composition with lung function and arterial stiffness parameters among young and elderly subjects.

		FEV1	FVC	FEV1 (%pred)	FVC (%pred)	AIx	PWV
**Young**	SDI	−0.362 (0.082)	**−0.411** (0.031)*	**−0.546** (0.006)**	**−0.573** (0.003)**	−0.068 (0.769)	0.132 (0.569)
	ISI	−0.396 (0.055)	**−0.460** (0.024)*	**−0.440** (0.032)*	**−0.482** (0.017)*	0.009 (0.967)	0.169 (0.465)
	Firmicutes	0.318 (0.130)	0.390 (0.060)	0.363 (0.081)	**0.409** (0.047)*	0.159 (0.492)	0.160 (0.489)
	Gemella	0.082 (0.704)	0.017 (0937)	−0.348 (0.095)	**−0.422** (0.040)*	−0.072 (0.756)	−0.080 (0.732)
	Actinobacteria	−0.055 (0.799)	−0.070 (0.747)	0.064 (0.767)	0.023 (0.916)	0.019 (0.935)	−0.121 (0.603)
	Actinomyces	**−0.408** (0.048)*	−0.391 (0.059)	−0.198 (0.354)	−0.142 (0.508)	−0.119 (0.608)	0.205 (0.372)
	Fusobacteria	−0.151 (0.480)	−0.168 (0.432)	−0.395 (0.056)	−0.364 (0.080)	0.014 (0.953)	0.146 (0.528)
	Leptotrichia	−0.132 (0.538)	−0.112 (0.603)	−0.353 (0.090)	−0.216 (0.310)	0.012 (0.960)	0.170 (0.461)
	Lautropia	0.043 (0.843)	−0.124 (0.563)	0.015 (0.944)	−0.209 (0.327)	**−0.492** (0.023)*	−0.420 (0.058)
**Elderly**	SDI	−0.151 (0.493)	−0.157 (0.475)	−0.155 (0.481)	−0.086 (0.696)	0.035 (0.870)	**−0.516** (0.010)*
	ISI	−0.216 (0.323)	−0.206 (0.346)	−0.112 (0.611)	−0.017 (0.939)	0.055 (0.789)	**−0.568** (0.004)**
	Firmicutes	0.190 (0.386)	0.099 (0.653)	−0.031 (0.888)	−0.200 (0.360)	0.193 (0.366)	0.118 (0.582)
	Gemella	−0.259 (0.232)	−0.215 (0.324)	−0.032 (0.887)	0.082 (0.710)	0.124 (0.564)	0.280 (0.186)
	Actinobacteria	0.347 (0.105)	**0.447** (0.033)*	0.382 (0.072)	0.322 (0.134)	0.137 (0.522)	0.097 (0.651)
	Actinomyces	0.230 (0.290)	0.238 (0.274)	0.124 (0.572)	0.073 (0.742)	0.023 (0.915)	−0.350 (0.094)
	Fusobacteria	−0.361 (0.091)	−0.344 (0.108)	−0.286 (0.187)	−0.114 (0.605)	0.083 (0.701)	**−0.570** (0.004)**
	Leptotrichia	−0.284 (0.189)	−0.221 (0.310)	−0.318 (0.139)	−0.157 (0.473)	0.207 (0.332)	**−0.569** (0.004)**
	Lautropia	−0.088 (0.690)	−0.112 (0.612)	0.254 (0.242)	0.080 (0.716)	0.034 (0.873)	−0.093 (0.665)

Microbiome indices of alpha diversity including the Shannon diversity index (SDI) and inverse Simpson index (ISI) as well as specific phyla and genera showing significant correlation with lung function (FEV1, FVC, FEV1%predicted, FVC %predicted) and arterial stiffness (augmentation index (AIx) and pulse wave velocity (PWV)) are displayed for younger and elderly sub-groups. *p* values are shown in brackets. Significantly correlation scores are indicated by bold typeface.

*p < 0.05,

**p < 0.01.

#### Age-dependent association of lung microbiome composition and lung function

**Fig 4:**
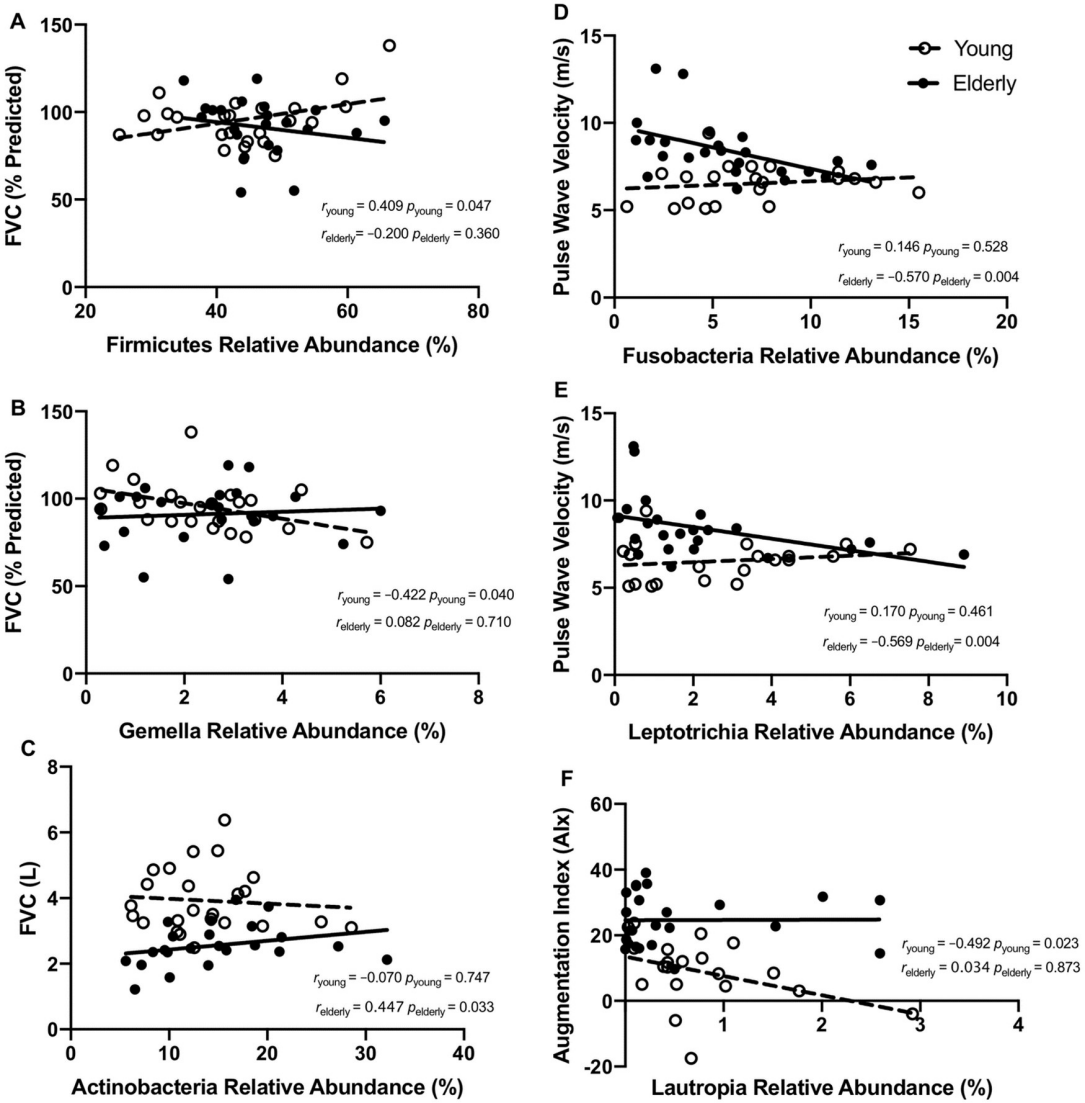
Physiological correlates of the microbiome at phylum and genus level. Sub-group correlations between specific lung microbiota, lung function and arterial stiffness parameters are indicated. Lung function—microbiome correlations were observed in the young but not the elderly group, between FVC (%Predicted) and relative abundance of (A) phylum Firmicutes and (B) genus *Gemella*. Correlations observed in the elderly but not the young group included (C) FVC and relative abundance of the genus *Actinobacteria*. Arterial stiffness and lung microbiome correlations were observed in the elderly group, between pulse wave velocity and relative abundance of (D) phylum Fusobacteria and (E) genus *Leptotrichia* while correlation between (F) augmentation index and the relative abundance of *Lautropia* was observed in young subjects. Young and elderly measures are indicated by unfilled and filled circles respectively. The line of best fit is indicated with a broken line for the young population and with a continuous line for the elderly. Correlation coefficients and associated *p* values are indicated for young sub-group (*r*_young_) and elderly sub-groups (*r*_elderly_).

We found that within the younger cohort, the relative abundance of microbes from the Firmicutes phylum (*r* = 0.409, *p =* 0.047) and specifically the *Gemella* genus (*r* = –0.422, *p =* 0.04) was associated with FVC (%Predicted) (Fig 4A and 4B). However, similar to correlations with SDI, these associations between FVC (%Predicted) were seen in the younger but not the elderly cohort (*r* = –0.200, *p =* 0.360 and *r* = 0.422, *p =* 0.710) (Fig 4A and 4B). In younger subjects, the relative abundance of *Actinomyces* genus was also inversely associated with absolute FEV1 but this association was not observed among older individuals (*r* = –0.408, *p =* 0.048 *vs*. *r* = 0.230, *p* = 0.290) (Table 2). Within the older group, the only association with lung function parameters was the relative abundance of phylum Actinobacteria, which was positively associated with absolute FVC (*r* = 0.447, *p =* 0.033), an association that was not observed in the younger group (*r* = –0.070, *p =* 0.747) (Fig 4C).

#### Age-dependent association of lung microbiome composition and arterial stiffness

**Fig 5:**
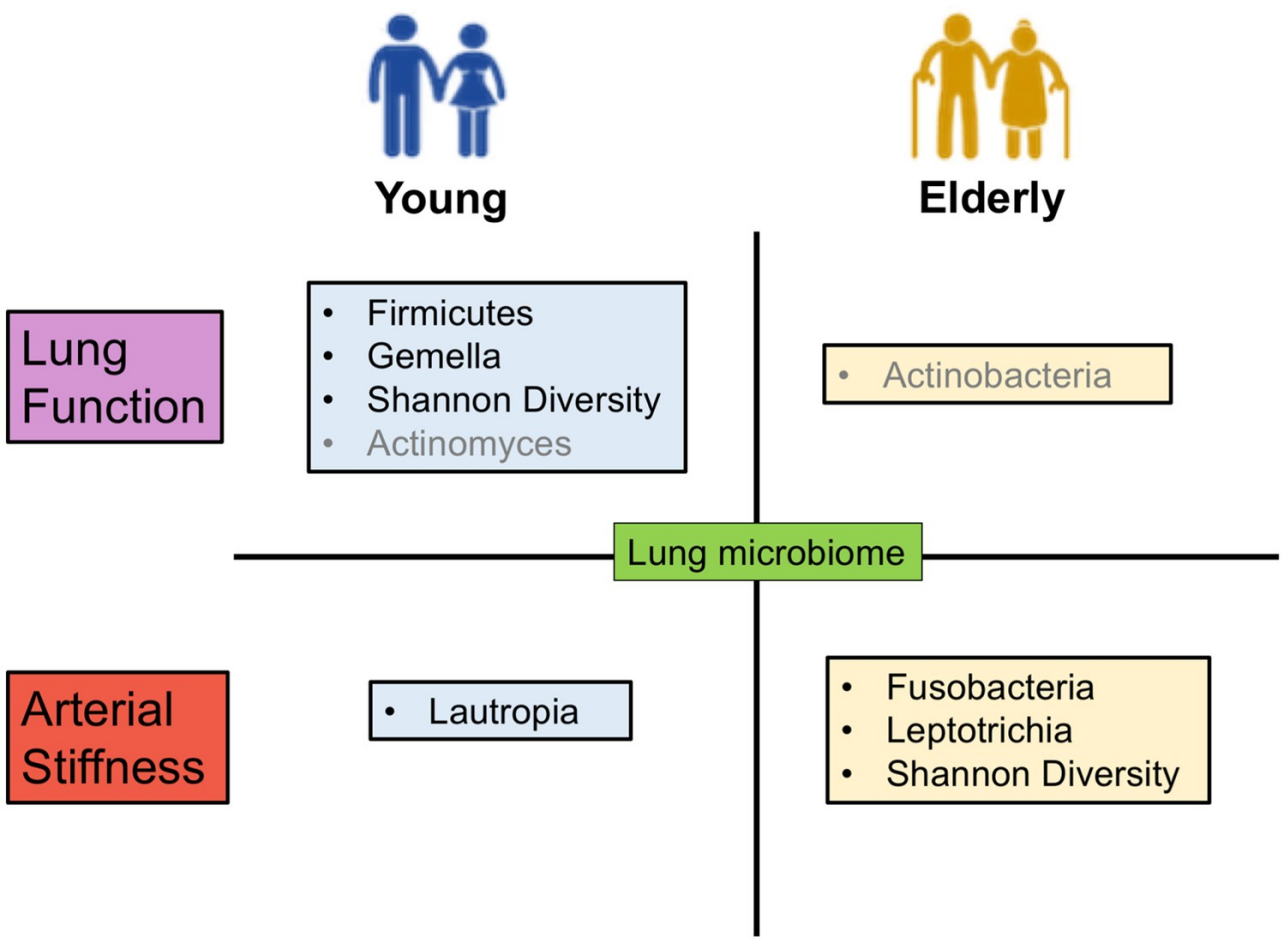
Summary schematic showing the significant associations between lung microbiome with lung function and arterial stiffness, within the young and elderly groups. Lung microbiome is associated with lung function mostly in the young group, as Shannon diversity, Firmicutes phylum and its genus *Gemella* are associated with predicted lung function, while absolute FEV1 is associated with *Actinomyces* (in grey). In the elderly group, absolute FVC is associated with Actinobacteria (in grey). Lung microbiome is associated with arterial stiffness mostly in the elderly group, with Shannon diversity, Fusobacteria phylum and its genus *Leptotrichia* associated with pulse wave velocity. In the young group, *Lautropia* is associated with arterial stiffness parameter augmentation index.

In contrast to the associations between lung microbiome and lung function, which were more prevalent in the younger group, the associations between lung microbiome and arterial stiffness were more pronounced in the older group. Within the older group, the arterial stiffness parameter PWV exhibited a strong inverse association with relative abundances of the Fusobacteria (*r* = –0.570, *p =* 0.004), specifically the genus *Leptotrichia* (*r* = –0.569, *p =* 0.004) (Fig 4D and 4E). These associations of PWV with both Fusobacteria and *Leptotrichia* relative abundances were not significant in the younger group (*r* = 0.146, *p =* 0.528 and *r* = 0.170, *p =* 0.461) (Fig 4D and 4E). The only genus exhibiting correlation with arterial stiffness in the young was *Lautropia*, which was associated with AIx an observation that was not evident in the older cohort (*r* = –0.492, *p =* 0.023 *vs r* = 0.034, *p =* 0.873) (Fig 4F). These age-specific associations between airway microbiome composition, lung function and arterial stiffness are summarised in Fig 5.

## Discussion

While a growing number of studies detail the ageing gut microbiome and its consequence for health and disease, comparable analyses of the respiratory microbiome are lacking [19, 40, 41]. Although several studies have investigated the airway microbiome in the context of respiratory disease, the present study is the first to explore age-associated differences in lung microbiome composition, with specific focus on healthy individuals of Asian origin. Our findings demonstrate remarkable consistency between young and elderly healthy microbiome profiles while identifying subtle taxonomic differences related to lung function and arterial stiffness in an age-dependent manner. These include associations between the lung microbiome and lung function (FEV1%Predicted and FVC %Predicted) in the younger adults, and arterial stiffness (PWV) in the elderly, suggesting that these relationships have greater relevance at different stages of life.

Given the paucity of evidence, the role of the airway microbiome in ageing is still unknown though long-speculated to be of clinical significance [42, 43]. Here, we have explored the effects of ageing on microbial constituents of the healthy airway microbiome by employing a paired genetic matching strategy, which controls the confounding influence of host genetics. Among patients with chronic respiratory disease such as COPD, overrepresentation of the Proteobacteria and reduction of Firmicute abundance is observed [44–47]. Enrichment of specific Proteobacteria including *Haemophilus* in the sputum microbiome of patients with COPD [48], and *Lautropia* in cystic fibrosis patients represent examples of disease-associated dysbiosis [46, 49]. In our healthy participants, however, the overall representation of phylum Proteobacteria (16%) was much lower than in disease states (~44%) [44]. Likewise, the average Firmicutes representation in our study (46%) was higher compared to diseased states (~16%) [44]. It is plausible that ‘healthy’ ageing of the lungs promotes enrichment of the Firmicutes and reduced relative abundance of Proteobacteria such as *Haemophilus* and *Lautropia*, which contrasted with the dysbiosis associated with chronic respiratory diseases.

We observed correlation between microbiome composition and lung function among younger subjects, noting an inverse relationship between lung microbiome diversity (SDI) and lung function (FEV1% predicted and FVC % predicted). Microbial diversity thus negatively correlated with lung function in our study, which contrasted with respiratory disease states where positive correlation is generally observed [50, 51]. At the phylum level, overall Firmicutes abundance positively associated with lung function (FVC %Predicted) in the younger adults in this study. This suggests enrichment of the Firmicutes has a beneficial effect on lung function and might explain the negative correlation between lung function and SDI. The increased Firmicute abundance, or that of other beneficial microbiome, could drive a corresponding reduction in SDI. Observed association between *Gemella* (a Firmicute linked to pulmonary exacerbation in cystic fibrosis patients) is also associated with reduced lung function in our young cohort, further suggesting its potentially negative implications for respiratory health [20, 52]. The positive influence of Firmicute abundance on lung function might be contingent on the presence and abundance of specific genera within this phylum, such as *Gemella*. The Actinobacteria (in the elderly) the genus *Actinomyces* (in the young) were associated with increased absolute FVC and decreased FEV1 respectively (Fig 5). However, absolute values of FEV1 and FVC are less robust lung function parameters that do not take into consideration normative physiological lung function decline with age. The association between lung microbiome and lung function parameters (FEV1 and FVC %Predicted) may thus be more relevant and pronounced in the younger group (Fig 5).

In contrast to lung function, we found that associations between airway microbiome composition and arterial stiffness were largely confined to older adults. Here, lung microbiome diversity was inversely associated with arterial stiffness parameter PWV (a negative clinical indicator), in the older adults. Reduced microbial diversity in lower airways is linked to inflammatory phenotypes of the airways [53–55], which could increase both systemic inflammation and arterial stiffness that occur during ageing [23, 48]. Our findings highlight the relative abundance of *Fusobacteria* phylum and its genus *Leptotrichia* in inverse association with arterial stiffness in older adults. The relevance of the Fusobacteria in lung respiratory disease has been noted, where they are associated with beneficial effects [56–58]. While associations between particular organisms and lung function occur mainly in the younger group, distinct organisms in the lung are linked to arterial stiffness in the elderly. These results highlight the complexity of the microbial interactions that may underpin age-dependent relationships between lung function and arterial stiffness across different stages of life (Fig 5).

Our study has several limitations. Although associations can be drawn from the study results, the cross-sectional design does not conclusively prove causality. This study is also unable to directly assess temporal dynamics of the lung microbiome during ageing given the lack of a longitudinal component. While the younger and older participants in this study are family pairs, which minimize genetic influences, we did not control for other confounders such as environmental factors, lifestyle or living conditions, that could have influenced the lung microbiome and represent significant confounders. As targeted bacterial 16s rRNA gene amplicon sequencing was employed, the resolution does not support species-level characterization of the microbiome, which could be important in understanding the ecological and functional interaction with ageing. Though our study was explorative in nature, it provides a first insight into age-dependant variability in the airway microbiome and its potential implications for respiratory and cardiovascular health. We have identified several potential microbial taxa whose clinical relevance should be further explicated in larger studies incorporating longitudinal experimental design to fully delineate the core healthy airway microbiome and the temporal dynamics of beneficial and deleterious taxa that may serve as early prognostic indicators of lung health status.

## Supporting information

S1 Fig Analysis of microbiome profiles of saliva and sputum. (A) Comparison of lung microbiome composition as detected by 16s rRNA gene profiling in DNA derived from saliva and sputum. Average relative abundance (RA) for the most representative taxa (present at >1%) is illustrated by circle size. Colour denotes phylum level membership. The saliva (V) and sputum (S) of three young (Y01-Y03) and three elderly matched family pairs (S01-S03) were analysed. (B) Principle co-ordinate analysis (PCoA) of Bray-Curtis distance between microbiome profiles observed in saliva samples (red open triangles) and sputum samples (black open circles) with indicated centroids (filled circles). Assessment of (C) Shannon diversity index and differences in relative abundance of (D) genus Granulicatella, (E) genus Leptotrichia and (F) genus Corynebacterium in saliva vs sputum samples among genetically paired young and elderly subjects. * = p < 0.05.
(TIF)

S2 Fig Alpha diversity indexes are shown between genetically paired young and elderly subjects. No significant differences are observed in (A) Shannon diversity index (B) Simpson index and (C) Inverse Simpson index between young (white bars) and elderly (grey bars) paired groups. Black connecting lines indicate young-elderly pairs. ns = not significant.
(TIF)

S1 Table Associations between lung function and arterial stiffness parameters, with and without adjustment for age in a partial correlation model. Correlation coefficients and corresponding *p* values are shown in brackets. *p < 0.05, **p < 0.01, ***p < 0.001. Significant correlation scores are indicated by bold typeface. AIx = Augmentation index, PWV = Pulse wave velocity.
(DOCX)

## References

[1] Budinger GRS, Kohanski RA, Gan W, Kobor MS, Amaral LA, Armanios M, et al The Intersection of Aging Biology and the Pathobiology of Lung Diseases: A Joint NHLBI/NIA Workshop. J Gerontol A Biol Sci Med Sci. 2017;72(11):1492–500. 10.1093/gerona/glx090 28498894 PMC5861849

[2] Benjamin EJ, Blaha MJ, Chiuve SE, Cushman M, Das SR, Deo R, et al Heart Disease and Stroke Statistics-2017 Update: A Report From the American Heart Association. Circulation. 2017;135(10):e146–e603. 10.1161/CIR.0000000000000485 28122885 PMC5408160

[3] Sharma G, Goodwin J. Effect of aging on respiratory system physiology and immunology. Clin Interv Aging. 2006;1(3):253–60. 18046878 10.2147/ciia.2006.1.3.253 PMC2695176

[4] Janssens JP, Pache JC, Nicod LP. Physiological changes in respiratory function associated with ageing. Eur Respir J. 1999;13(1):197–205. 10.1034/j.1399-3003.1999.13a36.x 10836348

[5] Lakatta EG, Levy D. Arterial and cardiac aging: major shareholders in cardiovascular disease enterprises: Part I: aging arteries: a "set up" for vascular disease. Circulation. 2003;107(1):139–46. 10.1161/01.cir.0000048892.83521.58 12515756

[6] Mitchell GF, Parise H, Benjamin EJ, Larson MG, Keyes MJ, Vita JA, et al Changes in arterial stiffness and wave reflection with advancing age in healthy men and women: the Framingham Heart Study. Hypertension. 2004;43(6):1239–45. 10.1161/01.HYP.0000128420.01881.aa 15123572

[7] Determinants of pulse wave velocity in healthy people and in the presence of cardiovascular risk factors: ‘establishing normal and reference values'. Eur Heart J. 2010;31(19):2338–50. 10.1093/eurheartj/ehq165 20530030 PMC2948201

[8] Jankowich MD, Taveira T, Wu WC. Decreased lung function is associated with increased arterial stiffness as measured by peripheral pulse pressure: data from NHANES III. Am J Hypertens. 2010;23(6):614–9. 10.1038/ajh.2010.37 20224559

[9] Zureik M, Benetos A, Neukirch C, Courbon D, Bean K, Thomas F, et al Reduced pulmonary function is associated with central arterial stiffness in men. Am J Respir Crit Care Med. 2001;164(12):2181–5. 10.1164/ajrccm.164.12.2107137 11751184

[10] Barr RG, Ahmed FS, Carr JJ, Hoffman EA, Jiang R, Kawut SM, et al Subclinical atherosclerosis, airflow obstruction and emphysema: the MESA Lung Study. Eur Respir J. 2012;39(4):846–54. 10.1183/09031936.00165410 22034646 PMC3616898

[11] Jacobs DR Jr., Yatsuya H, Hearst MO, Thyagarajan B, Kalhan R, Rosenberg S, et al Rate of decline of forced vital capacity predicts future arterial hypertension: the Coronary Artery Risk Development in Young Adults Study. Hypertension. 2012;59(2):219–25. 10.1161/HYPERTENSIONAHA.111.184101 22203738 PMC3269403

[12] Bolton CE, Cockcroft JR, Sabit R, Munnery M, McEniery CM, Wilkinson IB, et al Lung function in mid-life compared with later life is a stronger predictor of arterial stiffness in men: the Caerphilly Prospective Study. Int J Epidemiol. 2009;38(3):867–76. 10.1093/ije/dyn374 19204008

[13] Engstrom G, Lind P, Hedblad B, Wollmer P, Stavenow L, Janzon L, et al Lung function and cardiovascular risk: relationship with inflammation-sensitive plasma proteins. Circulation. 2002;106(20):2555–60. 10.1161/01.cir.0000037220.00065.0d 12427651

[14] Zureik M, Kauffmann F, Touboul PJ, Courbon D, Ducimetiere P. Association between peak expiratory flow and the development of carotid atherosclerotic plaques. Arch Intern Med. 2001;161(13):1669–76. 10.1001/archinte.161.13.1669 11434800

[15] Polverino F, Celli BR, Owen CA. COPD as an endothelial disorder: endothelial injury linking lesions in the lungs and other organs? (2017 Grover Conference Series). Pulm Circ. 2018;8(1):2045894018758528 10.1177/2045894018758528 29468936 PMC5826015

[16] Mills NL, Miller JJ, Anand A, Robinson SD, Frazer GA, Anderson D, et al Increased arterial stiffness in patients with chronic obstructive pulmonary disease: a mechanism for increased cardiovascular risk. Thorax. 2008;63(4):306–11. 10.1136/thx.2007.083493 18024535

[17] Qvist L, Nilsson U, Johansson V, Larsson K, Ronmark E, Langrish J, et al Central arterial stiffness is increased among subjects with severe and very severe COPD: report from a population-based cohort study. Eur Clin Respir J. 2015;2. 10.3402/ecrj.v2.27023 PMC4629768 26557263

[18] van Rooyen Y, Schutte AE, Huisman HW, Eloff FC, Du Plessis JL, Kruger A, et al Inflammation as Possible Mediator for the Relationship Between Lung and Arterial Function. Lung. 2016;194(1):107–15. 10.1007/s00408-015-9804-9 26411588

[19] Dickson RP, Huffnagle GB. The Lung Microbiome: New Principles for Respiratory Bacteriology in Health and Disease. PLoS Pathog. 2015;11(7):e1004923 10.1371/journal.ppat.1004923 26158874 PMC4497592

[20] Coburn B, Wang PW, Diaz Caballero J, Clark ST, Brahma V, Donaldson S, et al Lung microbiota across age and disease stage in cystic fibrosis. Sci Rep. 2015;5:10241 10.1038/srep10241 25974282 PMC4431465

[21] Dickson RP, Erb-Downward JR, Freeman CM, McCloskey L, Beck JM, Huffnagle GB, et al Spatial Variation in the Healthy Human Lung Microbiome and the Adapted Island Model of Lung Biogeography. Ann Am Thorac Soc. 2015;12(6):821–30. 10.1513/AnnalsATS.201501-029OC 25803243 PMC4590020

[22] Bassis CM, Erb-Downward JR, Dickson RP, Freeman CM, Schmidt TM, Young VB, et al Analysis of the upper respiratory tract microbiotas as the source of the lung and gastric microbiotas in healthy individuals. MBio. 2015;6(2):e00037 10.1128/mBio.00037-15 25736890 PMC4358017

[23] Erb-Downward JR, Thompson DL, Han MK, Freeman CM, McCloskey L, Schmidt LA, et al Analysis of the lung microbiome in the "healthy" smoker and in COPD. PLoS One. 2011;6(2):e16384 10.1371/journal.pone.0016384 21364979 PMC3043049

[24] Biagi E, Nylund L, Candela M, Ostan R, Bucci L, Pini E, et al Through ageing, and beyond: gut microbiota and inflammatory status in seniors and centenarians. PLoS One. 2010;5(5):e10667 10.1371/journal.pone.0010667 20498852 PMC2871786

[25] Claesson MJ, Jeffery IB, Conde S, Power SE, O’Connor EM, Cusack S, et al Gut microbiota composition correlates with diet and health in the elderly. Nature. 2012;488(7410):178–84. 10.1038/nature11319 22797518

[26] Hooper LV, Littman DR, Macpherson AJ. Interactions between the microbiota and the immune system. Science. 2012;336(6086):1268–73. 10.1126/science.1223490 22674334 PMC4420145

[27] El Assar M, Angulo J, Vallejo S, Peiro C, Sanchez-Ferrer CF, Rodriguez-Manas L. Mechanisms involved in the aging-induced vascular dysfunction. Front Physiol. 2012;3:132 10.3389/fphys.2012.00132 22783194 PMC3361078

[28] Mitchell GF, DeStefano AL, Larson MG, Benjamin EJ, Chen MH, Vasan RS, et al Heritability and a genome-wide linkage scan for arterial stiffness, wave reflection, and mean arterial pressure: the Framingham Heart Study. Circulation. 2005;112(2):194–9. 10.1161/CIRCULATIONAHA.104.530675 15998672

[29] Gursli S, Sandvik L, Bakkeheim E, Skrede B, Stuge B. Evaluation of a novel technique in airway clearance therapy—Specific Cough Technique (SCT) in cystic fibrosis: A pilot study of a series of N-of-1 randomised controlled trials. SAGE Open Med. 2017;5:2050312117697505 10.1177/2050312117697505 28540046 PMC5433674

[30] Fan X, Peters BA, Min D, Ahn J, Hayes RB. Comparison of the oral microbiome in mouthwash and whole saliva samples. PLoS One. 2018;13(4):e0194729 10.1371/journal.pone.0194729 29641531 PMC5894969

[31] Ma WY, Yang CY, Shih SR, Hsieh HJ, Hung CS, Chiu FC, et al Measurement of Waist Circumference: midabdominal or iliac crest? Diabetes Care. 2013;36(6):1660–6. 10.2337/dc12-1452 23275359 PMC3661855

[32] Pellegrino R, Viegi G, Brusasco V, Crapo RO, Burgos F, Casaburi R, et al Interpretative strategies for lung function tests. Eur Respir J. 2005;26(5):948 10.1183/09031936.05.00035205 16264058

[33] Nakagomi A, Shoji T, Okada S, Ohno Y, Kobayashi Y. Validity of the augmentation index and pulse pressure amplification as determined by the SphygmoCor XCEL device: a comparison with invasive measurements. Hypertens Res. 2018;41(1):27–32. 10.1038/hr.2017.81 28978987

[34] Sutton-Tyrrell K, Najjar SS, Boudreau RM, Venkitachalam L, Kupelian V, Simonsick EM, et al Elevated aortic pulse wave velocity, a marker of arterial stiffness, predicts cardiovascular events in well-functioning older adults. Circulation. 2005;111(25):3384–90. 10.1161/CIRCULATIONAHA.104.483628 15967850

[35] Van Bortel LM, Laurent S, Boutouyrie P, Chowienczyk P, Cruickshank JK, De Backer T, et al Expert consensus document on the measurement of aortic stiffness in daily practice using carotid-femoral pulse wave velocity. J Hypertens. 2012;30(3):445–8. 10.1097/HJH.0b013e32834fa8b0 22278144

[36] Mac Aogain M, Chandrasekaran R, Lim AYH, Low TB, Tan GL, Hassan T, et al Immunological corollary of the pulmonary mycobiome in bronchiectasis: the CAMEB study. Eur Respir J. 2018;52(1). 10.1183/13993003.00766-2018 PMC6092680 29880655

[37] Amplicon PCR, Clean-up, P.C.R. & Index, P.C.R. 16S Metagenomic Sequencing Library Preparation. https://www.illumina.com/content/dam/illumina-support/documents/documentation/chemistry_documentation/16s/16s-metagenomic-library-prep-guide-15044223-b.pdf.

[38] Davis NM, Proctor DM, Holmes SP, Relman DA, Callahan BJ. Simple statistical identification and removal of contaminant sequences in marker-gene and metagenomics data. Microbiome. 2018;6(1):226 10.1186/s40168-018-0605-2 30558668 PMC6298009

[39] Qaseem A, Wilt TJ, Weinberger SE, Hanania NA, Criner G, van der Molen T, et al Diagnosis and Management of Stable Chronic Obstructive Pulmonary Disease: A Clinical Practice Guideline Update from the American College of Physicians, American College of Chest Physicians, American Thoracic Society, and European Respiratory Society. Ann Intern Med. 2011;155(3):179–91. 10.7326/0003-4819-155-3-201108020-00008 21810710

[40] Seidel J, Valenzano DR. The role of the gut microbiome during host ageing. F1000Res. 2018;7. 10.12688/f1000research.15121.1 PMC6051225 30057748

[41] O’Toole PW, Jeffery IB. Gut microbiota and aging. Science. 2015;350(6265):1214–5. 10.1126/science.aac8469 26785481

[42] Murray MA, Chotirmall SH. The Impact of Immunosenescence on Pulmonary Disease. Mediators Inflamm. 2015;2015:692546 10.1155/2015/692546 26199462 PMC4495178

[43] Chotirmall SH, Burke CM. Aging and the microbiome: implications for asthma in the elderly? Expert Rev Respir Med. 2015;9(2):125–8. 10.1586/17476348.2015.1002473 25582135

[44] Garcia-Nunez M, Millares L, Pomares X, Ferrari R, Perez-Brocal V, Gallego M, et al Severity-related changes of bronchial microbiome in chronic obstructive pulmonary disease. J Clin Microbiol. 2014;52(12):4217–23. 10.1128/JCM.01967-14 25253795 PMC4313290

[45] Liu HY, Zhang SY, Yang WY, Su XF, He Y, Zhou HW, et al Oropharyngeal and Sputum Microbiomes Are Similar Following Exacerbation of Chronic Obstructive Pulmonary Disease. Front Microbiol. 2017;8:1163 10.3389/fmicb.2017.01163 28690603 PMC5479893

[46] Wang Z, Bafadhel M, Haldar K, Spivak A, Mayhew D, Miller BE, et al Lung microbiome dynamics in COPD exacerbations. Eur Respir J. 2016;47(4):1082–92. 10.1183/13993003.01406-2015 26917613

[47] Leung JM, Tiew PY, Mac Aogain M, Budden KF, Yong VF, Thomas SS, et al The role of acute and chronic respiratory colonization and infections in the pathogenesis of COPD. Respirology. 2017;22(4):634–50. 10.1111/resp.13032 28342288 PMC7169176

[48] Sze MA, Dimitriu PA, Suzuki M, McDonough JE, Campbell JD, Brothers JF, et al Host Response to the Lung Microbiome in Chronic Obstructive Pulmonary Disease. Am J Respir Crit Care Med. 2015;192(4):438–45. 10.1164/rccm.201502-0223OC 25945594 PMC4595667

[49] Huang YJ, Boushey HA. The Sputum Microbiome in Chronic Obstructive Pulmonary Disease Exacerbations. Ann Am Thorac Soc. 2015;12 Suppl 2:S176–80. 26595736 10.1513/AnnalsATS.201506-319AW PMC4722839

[50] O’Dwyer DN, Dickson RP, Moore BB. The Lung Microbiome, Immunity, and the Pathogenesis of Chronic Lung Disease. J Immunol. 2016;196(12):4839–47. 10.4049/jimmunol.1600279 27260767 PMC4894335

[51] Budden KF, Shukla SD, Rehman SF, Bowerman KL, Keely S, Hugenholtz P, et al Functional effects of the microbiota in chronic respiratory disease. Lancet Respir Med. 2019. 10.1016/S2213-2600(18)30510-1 30975495

[52] Carmody LA, Zhao J, Schloss PD, Petrosino JF, Murray S, Young VB, et al Changes in cystic fibrosis airway microbiota at pulmonary exacerbation. Ann Am Thorac Soc. 2013;10(3):179–87. 10.1513/AnnalsATS.201211-107OC 23802813 PMC3960905

[53] Takahashi Y, Saito A, Chiba H, Kuronuma K, Ikeda K, Kobayashi T, et al Impaired diversity of the lung microbiome predicts progression of idiopathic pulmonary fibrosis. Respir Res. 2018;19(1):34 10.1186/s12931-018-0736-9 29486761 PMC6389110

[54] Flight WG, Smith A, Paisey C, Marchesi JR, Bull MJ, Norville PJ, et al Rapid Detection of Emerging Pathogens and Loss of Microbial Diversity Associated with Severe Lung Disease in Cystic Fibrosis. J Clin Microbiol. 2015;53(7):2022–9. 10.1128/JCM.00432-15 25878338 PMC4473198

[55] Jorth P, Ehsan Z, Rezayat A, Caldwell E, Pope C, Brewington JJ, et al Direct Lung Sampling Indicates That Established Pathogens Dominate Early Infections in Children with Cystic Fibrosis. Cell Rep. 2019;27(4):1190–204.e3. 10.1016/j.celrep.2019.03.086 31018133 PMC6668708

[56] Tuleta I, Skowasch D, Aurich F, Eckstein N, Schueler R, Pizarro C, et al Asthma is associated with atherosclerotic artery changes. PLoS One. 2017;12(10):e0186820 10.1371/journal.pone.0186820 29073174 PMC5658104

[57] Steinmann M, Abbas C, Singer F, Casaulta C, Regamey N, Haffner D, et al Arterial stiffness is increased in asthmatic children. Eur J Pediatr. 2015;174(4):519–23. 10.1007/s00431-014-2423-2 25248341

[58] Hosgood HD 3rd, Mongodin EF, Wan Y, Hua X, Rothman N, Hu W, et al The respiratory tract microbiome and its relationship to lung cancer and environmental exposures found in rural China. Environ Mol Mutagen. 2019. 10.1002/em.22291 PMC8259386 30942501

