# Supplementary figures and images for: Airway microbiome composition correlates with lung function and arterial stiffness in an age-dependent manner
Source: PLoS One. 2019 Nov 26;14(11):e0225636. doi: 10.1371/journal.pone.0225636 (PMC6879132; doi:10.1371/journal.pone.0225636)

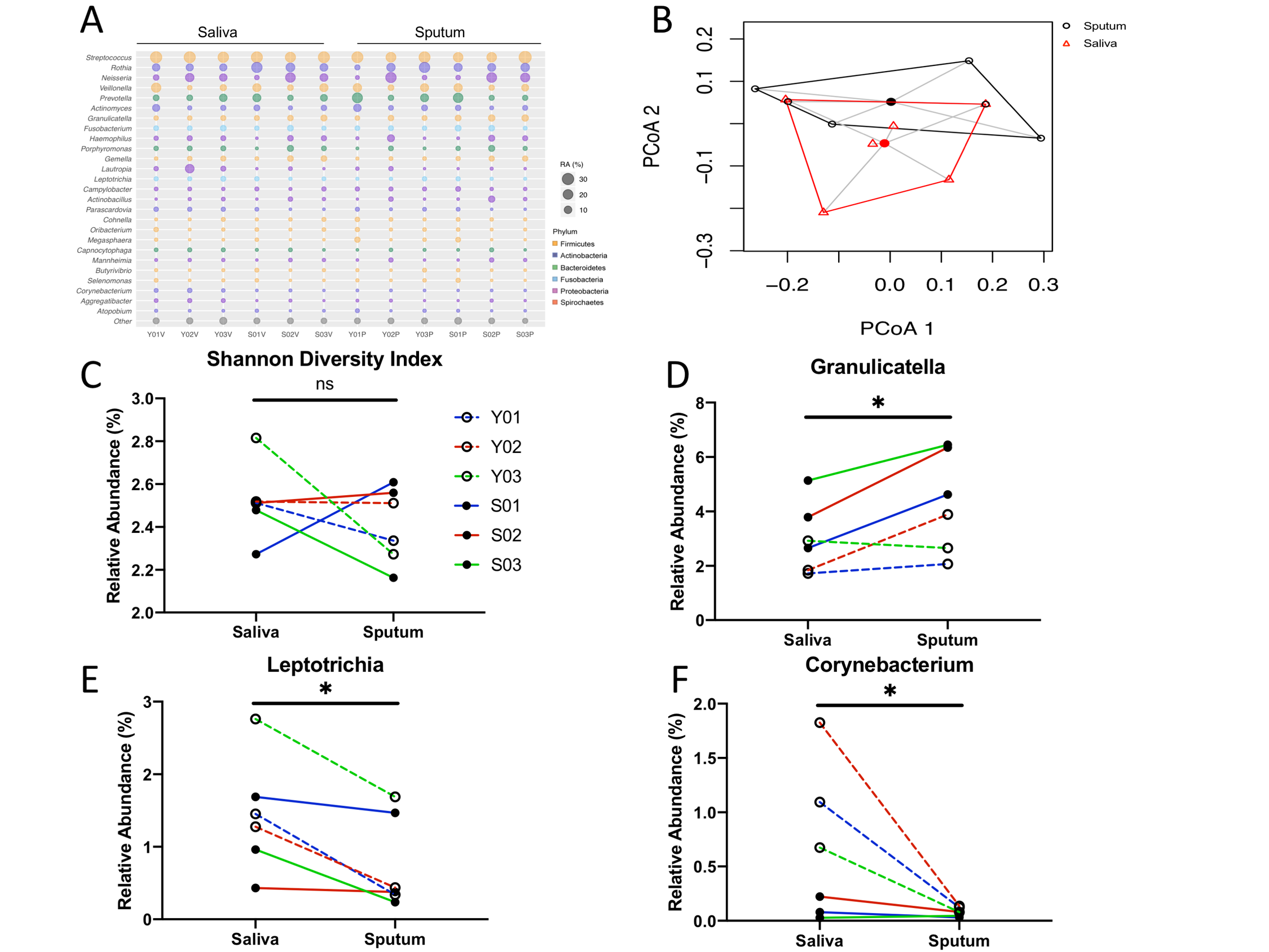

Supplement: S1 Fig — (A) Comparison of lung microbiome composition as detected by 16s rRNA gene profiling in DNA derived from saliva and sputum. Average relative abundance (RA) for the most representative taxa (present at >1%) is illustrated by circle size. Colour denotes phylum level membership. The saliva (V) and sputum (S) of three young (Y01-Y03) and three elderly matched family pairs (S01-S03) were analysed. (B) Principle co-ordinate analysis (PCoA) of Bray-Curtis distance between microbiome profiles observed in saliva samples (red open triangles) and sputum samples (black open circles) with indicated centroids (filled circles). Assessment of (C) Shannon diversity index and differences in relative abundance of (D) genus Granulicatella, (E) genus Leptotrichia and (F) genus Corynebacterium in saliva vs sputum samples among genetically paired young and elderly subjects. * = p < 0.05. (TIF) [file pone.0225636.s001.tif]

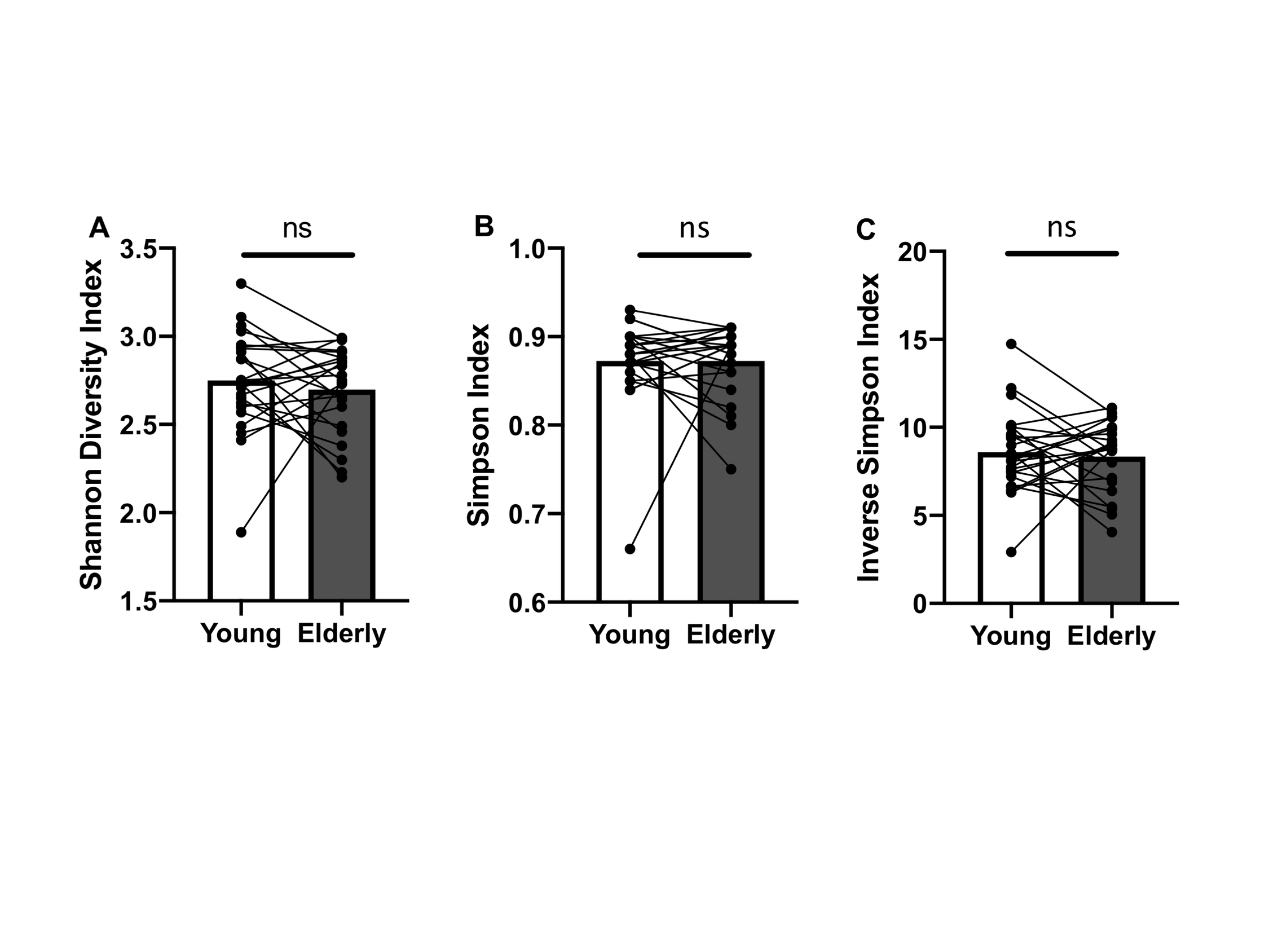

Supplement: S2 Fig — No significant differences are observed in (A) Shannon diversity index (B) Simpson index and (C) Inverse Simpson index between young (white bars) and elderly (grey bars) paired groups. Black connecting lines indicate young-elderly pairs. ns = not significant. (TIF) [file pone.0225636.s002.tif]
