# Supplementary material for: Airway microbiome composition correlates with lung function and arterial stiffness in an age-dependent manner
Source: PLoS One. 2019 Nov 26;14(11):e0225636. doi: 10.1371/journal.pone.0225636 (PMC6879132; doi:10.1371/journal.pone.0225636)
Supplement: S1 Table — Correlation coefficients and corresponding p values are shown in brackets. *p < 0.05, **p < 0.01, ***p < 0.001. Significant correlation scores are indicated by bold typeface. AIx = Augmentation index, PWV = Pulse wave velocity. (DOCX) [file pone.0225636.s003.docx]

**S1 Table**

|  | **Without age adjustment** | | **With age adjustment** | |
| --- | --- | --- | --- | --- |
|  | **AIx** | **PWV** | **AIx** | **PWV** |
| FEV1 (L) | **–0.519**  **(<0.001)***** | **–0.396**  **(0.008)**** | –0.043  (0.786) | 0.098  (0.531) |
| FVC (L) | **–0.509**  **(<0.001)***** | **–0.321**  **(0.033)*** | –0.108  (0.492) | 0.165  (0.290) |
| FEV1/FVC (%) | –0.247  (0.106) | **–0.508**  **(<0.001)***** | 0.194  (0.212) | –0.158  (0313) |
| FEV1 (% Predicted) | –0.182  (0.237) | –0.118  (0.445) | –0.017  (0.913) | –0.027  (0.865) |
| FVC (% Predicted) | –0.129  (0.404) | 0.064  (0.681) | –0.121  (0.439) | 0.034  (0.831) |
| FEV1/FVC (% Predicted) | –0.054  (0.727) | –0.253  (0.098) | 0.190  (0.223) | –0.099  (0.526) |
